# Associations between expression of indoleamine 2, 3-dioxygenase enzyme and inflammatory cytokines in patients with first-episode drug-naive Schizophrenia

**DOI:** 10.1038/s41398-021-01688-x

**Published:** 2021-11-20

**Authors:** Yan Zhang, Han Shi, Ge Yang, Yongfeng Yang, Wenqiang Li, Meng Song, Minglong Shao, Xi Su, Luxian Lv

**Affiliations:** 1 Henan Mental Hospital, The Second Affiliated Hospital of Xinxiang Medical University, Xinxiang, China; 2 Henan Key Lab of Biological Psychiatry of Xinxiang Medical University, Xinxiang, China; 3 International Joint Research Laboratory for Psychiatry and Neuroscience of Henan, Xinxiang, China

**Keywords:** Schizophrenia, Predictive markers

## Abstract

The indoleamine 2,3-dioxygenase (IDO) enzyme is the first rate-limiting enzyme of the tryptophan degradation pathway in which dysfunction of neuroactive metabolites has been implicated in the pathophysiology of schizophrenia. Inflammatory molecules such as pro-inflammatory cytokines could enhance the activity of IDO. There are few studies on the expression of IDO levels and its correlation with levels of inflammatory cytokines in first-episode drug-naive patients with schizophrenia. One hundred inpatients (female = 33, male = 67) with first-episode drug-naive schizophrenia entered a 6-week, double-blind, randomized, placebo-controlled clinical trial. All individuals were assigned celecoxib or placebo combined with risperidone. Serum levels of IDO and six inflammatory cytokines (IL-1β, IL-6, TNF-α IL-17, IL-4, and INF-γ) were measured. The Positive and Negative Syndrome Scale (PANSS) was used to assess the severity of psychotic symptoms. Compared to healthy subjects, patients had significantly elevated levels of IDO and six cytokines at baseline. Over the 6-week treatment period, the decrease in the levels of IDO and TNF-α and the improvement in the PANSS total score, positive scores, and negative scores in the celecoxib group were significantly greater than in the placebo group. There was a significantly positive correlation between IDO levels and the PANSS negative scores and between IDO levels and TNF-α and IFN-γ levels in the celecoxib group. These findings showed abnormal expression of IDO levels which correlated with negative symptoms and pro-inflammatory cytokine levels in patients with first-episode drug-naive schizophrenia, suggesting the important role of IDO in the pathological mechanism of schizophrenia. Registration number: ChiCTR2000041403.

## Introduction

Schizophrenia is a common severe mental illness with a lifetime prevalence of about 1% worldwide [1]. Because of genetic mutations and environmental factors, the development of schizophrenia is a very complex process and is determined by multistage factors. Accumulating evidence suggests that immune dysfunction, caused by autoimmune reaction [2], viral infection [3], or macrophage-T-lymphocyte activation [4] plays an important role in the pathogenesis of schizophrenia.

The immune hypothesis presents a possible treatment for patients with schizophrenia. Pharmacological studies and animal experiments have found that some agents with anti-inflammatory properties, such as the anti-inflammatory drug minocycline, can improve psychotic symptoms [5–8]. The underlying mechanisms of immune dysregulation are associated with inflammation in the central nervous system (CNS), such as changes in the activity status of microglia cells [9]. Minocycline can cross the blood-brain barrier to suppress activated microglia cells, and ultimately improve the negative symptoms of schizophrenia [6, 10, 11]. Other studies have shown the potential anti-inflammatory properties of many psychotropic agents, such as antipsychotics, selective serotonin reuptake inhibitors, lithium, and valproate acid [3, 12]. Therefore, anti-inflammatory drugs are potential candidates as antipsychotic compounds. The cyclooxygenase-2 (COX-2) inhibitor celecoxib is a non-steroidal anti-inflammatory drug. COX-2 is an enzyme synthesizing PGE involved in inflammatory processes [13]. In vivo animal experiments have suggested that COX-2 inhibition can limit the increase in the production of the pro-inflammatory cytokines IL-1β and TNFα, as well that of PGE2 [14]. COX-2 inhibitors (mainly celecoxib) exhibited antipsychotic properties in vitro and in animal studies [15]. However, clinical trials of celecoxib as an adjunct to antipsychotics in the treatment of schizophrenia have been inconsistent [16–19]. These discordances may be related to a number of factors, such as sample size, disease state (acute versus chronic), and disease duration. Thus, compared to chronic or long-term medicated patients, drug-naive, first-episode schizophrenia patients are of unique value in exploring the neuropathological processes of the disease and in developing new drugs by minimizing potential confounders.

Tryptophan (Trp) is an essential amino acid and is necessary for cell survival and protein synthesis. The kynurenine pathway (KP) is quantitatively the most important, accounting for ~95% of Trp metabolism and involves several neuroactive metabolites. Indoleamine 2,3-dioxygenase (IDO) is the initial and rate-limiting enzyme in the KP and catalyzes Trp into N-formyl-l-kynurenine which is a precursor of some neuroactive metabolites such as kynurenic acid (KYNA), an endogenous antagonist of the N-methyl-d-aspartate (NMDA) receptor and a7 nicotinic acetylcholine receptor (a7nAChR) [20], and the NMDA receptor agonist quinolinic acid. When there is inflammation, infection, or oxidative stress, pro-inflammatory molecules such as cytokines and reactive oxygen species can enhance the activity of the enzyme IDO. Inhibition of the inflammatory process may impede IDO-mediated Trp catabolism [21]. For example, the pro-inflammatory cytokine interferon (IFN)-γ can potently induce both the enzymatic activity and gene expression of IDO [22]. Other pro-inflammatory cytokines, such as prostaglandin (PGE) 2 or tumor necrosis factor-alpha (TNF-α), induce an increase in IDO activity by acting in a synergistic manner with IFN-γ [23]. However, anti-inflammatory cytokines such as interleukin (IL)-4 inhibit IDO activity [24]. So far, studies on the correlation between inflammatory cytokines and IDO in psychiatric disorders has mainly focused on depression. Although there is no direct evidence, previous studies have found that increased IDO activity, induced by pro-inflammatory cytokines, can lead to the development of depression [25–27]. There are few studies on the correlation between IDO and cytokines in schizophrenia, and further studies are needed.

A previous study found that the COX-2 inhibitor celecoxib inhibits IDO-mediated immune tolerance through regulatory T cells [28]. In another study, celecoxib reverses the IFN-α-induced increase in the kynurenine/Trp ratio, indicative of IDO activation [27]. However, it is unclear whether celecoxib is involved in the regulation of IDO activity in schizophrenia.

The present study is a 6-week, randomized, double-blind, and placebo-controlled trial to investigate the expression of IDO in serum blood of first-episode drug-naive schizophrenia patients and to explore the correlation between IDO and various inflammatory cytokines by using the COX2 inhibitor celecoxib. Based on previously reported results, we hypothesized that there would be abnormally elevated IDO expression in the peripheral blood of patients with first-episode schizophrenia, and IDO expression levels would be correlated with the pro-inflammatory cytokine levels and the severity of psychiatric symptoms.

## Methods

### Design

This was a 6-week, randomized, double-blind, and placebo-controlled trial to investigate the expression of IDO in serum blood of first-episode drug-naive schizophrenia patients, and to explore the correlation between IDO and various inflammatory cytokines. The study protocol was approved by the Ethics Committee of The Second Affiliated Hospital of Xinxiang Medical University. Participant recruitment occurred from April 2019 to October 2020. All participants provided written informed consent. The trial is registered with ClinicalTrials.gov (ChiCTR2000041403).

### Subjects

A total of 113 inpatients with schizophrenia were recruited from the Department of Psychiatry of The Second Affiliated Hospital of Xinxiang Medical University. The inclusion criteria were: (1) aged 16‒55 years; (2) meeting the Diagnostic and Statistical Manual of Mental Disorders, Fourth Edition-Text Revision (DSM-IV-TR) criteria for schizophrenia; (3) score on the Positive and Negative Syndrome Scale (PANSS) > 60; (4) duration of disease less than 6 months; (5) antipsychotic-naïve or treatment duration < 2 weeks before study entry; (6) an intelligence quotient (IQ) of at least 70; (7) at least 6 years of formal education; and (8) Han Chinese ethnicity. Fifty healthy controls were recruited for this study from the local community through advertisements. Psychiatric conditions were ruled out in healthy controls using the Structured Clinical Interview for DSM Disorders (SCID). In addition, a thorough physical exam was completed by the same research psychiatrists to rule out any medical conditions. The exclusion criteria for all participants included: (1) a psychiatric diagnosis other than schizophrenia (determined by SCID); (2) comorbid serious or unstable medical conditions, or significant inflammatory or immune conditions, including heart disease, epilepsy, hepatic or renal diseases, diabetes, aplastic anemia, systemic lupus erythematous or asthma; (3) treatment with anti-inflammatory drugs (such as celecoxib, β-lactam antibiotics), hormones or immunosuppressant agents in the 6 months before study entry; (4) contraindication to celecoxib treatment; (5) planning to become pregnant, or were pregnant or breastfeeding.

### Randomization and masking

After screening, all individuals with first-episode drug-naive schizophrenia entered a 6-week, double blind, placebo-controlled study. All patients were randomized to celecoxib (200 mg/day) or placebo, as additional therapy to risperidone 4 mg to 6 mg/day, by a dynamic randomization method called minimization that equalizes the treatment groups across stratification variables such as gender and age. The random code was generated at the off-site hospital clinical drug trial base and sent to the off-site clinical trial pharmacy. To ensure concealment of the treatment assignment, randomization was conducted by a research pharmacist at a separate facility and medication was provided in coded containers of identical-appearing capsules of celecoxib or placebo, which were manufactured and dispensed by the same pharmaceutical manufacturer. To maintain blinding, all subjects took a capsule of 200 mg celecoxib or placebo every day depending on the randomization group. The dose of risperidone was gradually increased from 1 mg per day and adjusted from 4 mg to 6 mg/day according to the patient’s condition in 2 weeks. According to the needs of the disease, trihexyphenidyl and propranolol could be used to relieve the side effects of drugs, and benzodiazepine could be used to improve sleep. All study personnel, including participants, treating team, statistician, investigators, and assessors were blinded to treatment assignment for the study duration.

### Clinical measures

At baseline and 6 weeks of follow-up, PANSS scores were collected to record the treatment efficacy. Investigators completed training for these study scales and showed excellent inter-rater reliability and consistency on the scales (Kappa = 0.85).

### Measurement of serum IDO and cytokine levels

Serum levels of IDO and cytokines (TNF-α, IL-1β, IFN-γ, IL-4, IL-6, IL-17) were determined at baseline and at 6 weeks of follow-up. Venous blood was collected from the forearm between 6 and 7 a.m. following an overnight fast. Serum levels of IDO and six cytokines were measured by quantitative enzyme-linked immunosorbent assay (ELISA) using a commercially available kit: IDO (SEB547Hu, sensitivity: minimum detectable measurement ≤ 0.118 ng/mL) (Cloud-Clone, Wuhan, China), IFN-γ (SEA049Hu, sensitivity: minimum detectable measurement ≤ 6.1 pg/ml) (Cloud-Clone, Wuhan, China), IL-4 (SEA077Hu, sensitivity: minimum detectable measurement≤5.9 pg/ml) (Cloud-Clone, Wuhan, China), IL-6 (SEA079Hu, sensitivity: minimum detectable measurement ≤ 3.2 pg/ml) (Cloud-Clone, Wuhan, China), and IL-17 (SEA063Hu, sensitivity: 5.5 pg/ml) (Cloud-Clone, Wuhan, China), TNF-α (DTA00D, sensitivity: 6.23 pg/ml) (R&D Systems, USA), IL-1β (DLB50, sensitivity: 1 pg/ml) (R&D Systems, USA). All ELISA kits used in this study have been cross-reactivity validation of existing related factors to ensure their specificity. All assays were blinded to group or behavior information until after the results were finalized.

### Outcomes

The primary outcomes were circulating IDO levels at baseline and week 6. The secondary outcomes were serum levels of target cytokines and total score on the PANSS, all subscale scores on the PANSS at baseline and week 6.

### Statistical analysis

Statistical analysis was conducted using the SPSS software (version 23.0; SPSS Inc., Chicago, IL, USA). Descriptive statistics were performed to summarize the demographic and clinical characteristics of the study sample. Group comparisons were performed using analysis of variance (ANOVA) and independent *T*-test for continuous variables and Chi-square test for categorical variables. A paired sample *T*-test was used for intra-group comparison. A one-sample Kolmogorov–Smirnov test was used to examine each variable for normality. All data were tested to conform to normal distribution. Pearson’s correlation was used for the correlation analysis. For all analyses, a *P* value < 0.05 (2-tailed) was deemed statistically significant.

## Results

**Fig. 1:**
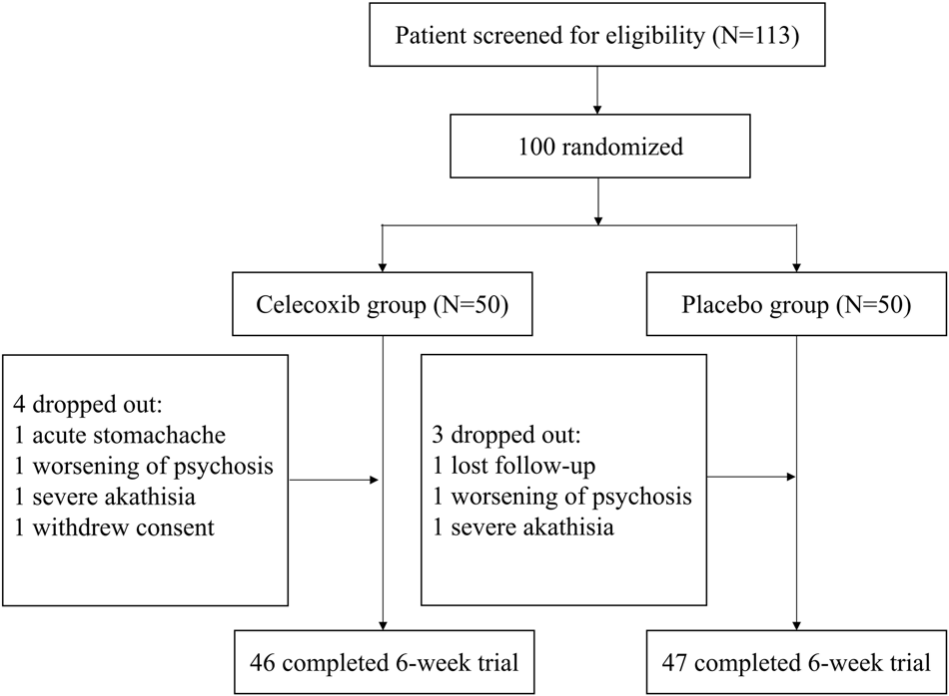
Flowchart of run-in and maintenance treatment of patients with first-episode drugnaive schizophrenia. IDO: indoleamine 2, 3-dioxygenase enzyme; celecoxib group: schizophrenia patients were treated with celecoxib combination with risperidone; placebo group: schizophrenia patients were treated with placebo combination with risperidone.

One hundred inpatients with first-episode drug-naive schizophrenia were screened for the study. These individuals were randomized to trial medication (50 patients in each group). Ninety-three patients (93%) completed the 6-week trial: 46 on celecoxib, 47 on placebo (see Fig. 1). Four patients in the celecoxib group and three patients in the placebo group dropped out before the end of the study. The dropouts from the celecoxib group were aged 26, 29, 35, and 40 years (two women, two men), and they dropped out at days 7, 23, 17, and 15, respectively. The reasons for dropping out were acute stomachache, worsening of psychosis, severe akathisia, and withdrawal of consent. The dropouts from the placebo group were aged 29, 31, and 43 years (one woman, two men), and they dropped out at days 6, 16, and 33, respectively. The reasons for dropping out were lost follow-up, worsening of psychosis, and severe akathisia. There were no significant differences among the three groups in terms of age, gender, or years of education. There was no significant difference in scores on the PANSS and on all subscales between the two treatment groups (see Table 1).

**Table 1:** Demographic characteristics and clinical characteristics and serum measures for different subgroups of patients and healthy groups.

Variable	Schizophrenia group		Healthy group (*n* = 50)	*F*/*T*/*Χ*^2^	*P*
	Celecoxib group (*n* = 46)	Placebo group (*n* = 47)			
Age(years)	30.46 ± 8.59	27.85 ± 6.97	29.96 ± 6.01	1.176	0.184
*Gender*					
Male	34 (73.9)	29 (61.7)	34 (68.0)	1.590	0.452
Female	12	18	16		
Education (years)	11.65 ± 2.72	10.96 ± 2.43	11.90 ± 2.91	1.566	0.212
PANSS total	90.17 ± 10.61	94.00 ± 10.06	–	−1.785	0.078
PANSS positive	23.96 ± 5.11	24.26 ± 3.08	–	−0.342	0.773
PANSS negative	23.09 ± 5.02	24.17 ± 4.14	–	−1.136	0.259
PANSS general	43.13 ± 7.17	45.57 ± 6.92	–	−1.672	0.098

Between-group differences were performed using ANOVA; Between-group differences were performed using independent sample *T*-test; Chi-square analysis was used for categorical variables.

**Fig. 2:**
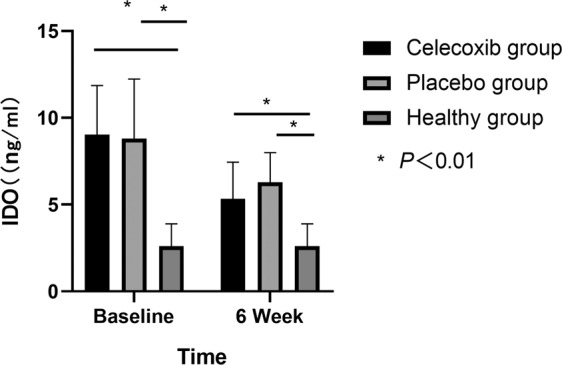
Treatment outcome of IDO for celecoxib and placebo groups. IDO: indoleamine 2, 3-dioxygenase enzyme; celecoxib group: schizophrenia patients were treated with celecoxib combination with risperidone; placebo group: schizophrenia patients were treated with placebo combination with risperidone.

The serum levels of IDO of the two treatment groups were significantly higher than that of the healthy group (*F* = 93.165, *P* = 0.000). There was no significant difference in the IDO levels between the two treatment groups (*P* = 0.683) (see Table 2). Over the 6-week treatment period, there was a significant difference in the decrease in serum levels of IDO in both treatment groups (*P* < 0.05). The decrease in serum levels of IDO in the celecoxib group was significantly greater than in the placebo group at 6 weeks (*t* = −2.383, *P* = 0.019) (see Table 2, Fig. 2).

**Table 2:** Treatment outcome of IDO for different subgroups of patients and healthy group.

Variable	Schizophrenia group		Healthy group (*n* = 50)	*F*/*T*	*P*	*P*1	*P*2	*P*3
	Celecoxib group (*n* = 46)	Placebo group (*n* = 47)						
*IDO (ng/ml)*								
Baseline	9.03 ± 2.81	8.81 ± 3.42	2.60 ± 1.29	93.165	0.000	0.000	0.000	0.683
6 week	5.34 ± 2.11^a^	6.29 ± 1.71^a^	–	−2.383	0.019			

Within-group differences were examined using paired *T*-test; Between-group differences were performed using ANOVA; Between-group differences were performed using independent sample *T*-test. *P*1: For between celecoxib group and healthy group; *P*2: For between placebo group and healthy group; *P*3: For between celecoxib group and placebo group.

^a^*P* < 0.05, for between the 6-week time point and baseline.

There was a significant improvement in scores on the PANSS and on all subscales over the 6 weeks of treatment in both groups of patients (all *P* < 0.001). The improvement in the PANSS total score, positive scores and negative scores in the celecoxib group was significantly greater than for the placebo group at 6 weeks (*t* = −3.618, *P* = 0.000; *t* = −3.622, *P* = 0.000; *t* = −3.255, *P* = 0.002) (see Table 3). No significant difference was observed in the improvement of the PANSS general score at 6 weeks between the two groups.

**Table 3:** Main clinical outcome measures for celecoxib and placebo groups.

Variable	Celecoxib group (*n* = 46)	Placebo group (*n* = 47)	*T*	*P*
*PANSS total*				
Baseline	90.17 ± 10.61	94.00 ± 10.06	−1.785	0.078
6 week	58.50 ± 12.35^**^	67.57 ± 11.84^**^	−3.618	0.000
*PANSS positive*				
Baseline	23.96 ± 5.11	24.26 ± 3.08	−0.340	0.735
6 week	15.76 ± 4.66^**^	19.43 ± 5.08^**^	−3.622	0.000
*PANSS negative*				
Baseline	23.09 ± 5.02	24.26 ± 3.09	−1.136	0.259
6 week	14.57 ± 4.41^**^	18.13 ± 6.01^**^	−3.255	0.002
*PANSS general*				
Baseline	43.13 ± 7.16	45.57 ± 6.92	−1.672	0.098
6 week	28.17 ± 6.28^**^	30.02 ± 5.71^**^	−1.484	0.141

Within-group differences were examined using paired *T*-test. Between-group differences were performed using independent sample *T*-test.

**P < 0.01, for between the 6-week time point and baseline.

**Fig. 3:**
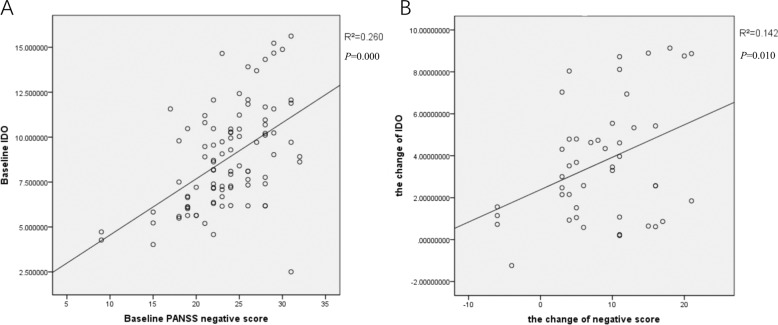
Correlation coefficients between IDO levels and PANSS negative score. **A** There was a significant correlation between serum IDO levels and the PANSS negative symptom scores in all schizophrenia patients. **B** The reduction in the PANSS negative score showed a significant positive correlation with the decrease in serum levels of IDO in the celecoxib group. *P* < 0.05.

In order to analyze the effect of celecoxib on psychotic symptoms, we conducted correlation analyses between serum levels of IDO and the scores on the PANSS and on all subscales at baseline, and between the change in serum levels of IDO and the change in scores on the PANSS and on all subscales over 6 weeks in the two treatment groups using Pearson’s correlation analysis. There was a significant correlation between serum IDO levels and the PANSS negative symptom scores in all schizophrenia patients (*r* = 0.260, *P* = 0.000) (see Fig. 3A). The reduction in the PANSS negative score showed a significant positive correlation with the decrease in serum levels of IDO in the celecoxib group (*r* = 0.142, *P* = 0.010) (see Fig. 3B).

The serum levels of TNF-α, IL-1β, IL-6, IL-17, IL-4, and IFN-γ in the two treatment groups were significantly higher than those of healthy volunteers at baseline (all *P* < 0.05), and there was no significant difference in the levels of inflammatory cytokines between the two treatment groups (see Table 4). Over the 6-week treatment period, there were significant differences in the decrease in serum levels of TNF-α, IFN-γ, and IL-17 in both groups (all *P* < 0.05). A significant decrease in serum IL-1β levels was observed in the celecoxib group at 6 weeks compared to baseline (*P* < 0.05). No significant difference was observed in the serum levels of IL-4 or IL-6 in the two treatment groups before and after treatment. The decrease in serum levels of TNF-α in the celecoxib group was significantly greater than in the placebo group at 6 weeks (*t* = −13.989, *P* = 0.000) (see Table 4).

**Table 4:** Treatment outcome of inflammatory cytokines for different subgroups of patients and healthy groups.

Variable	Schizophrenia group		Healthy group (*n* = 50)	*F*/*T*	*P*	*P*1	*P*2	*P*3
	Celecoxib group (*n* = 46)	Placebo group (*n* = 47)						
*TNF-α* *(pg/ml)*								
baseline	62.47 ± 0.57	62.67 ± 0.75	2.11 ± 0.59	143133.552	0.000	0.000	0.000	0.153
6 week	11.32 ± 0.19^**^	21.15 ± 4.81^**^	–	−13.989	0.000			
*IL-1β* *(pg/ml)*								
baseline	2.46 ± 0.55	2.28 ± 0.49	NA	1.656	0.101			
6 week	1.93 ± 0.33^**^	2.01 ± 0.34	–	−1.147	0.254			
*INF-γ (pg/ml)*								
baseline	448.79 ± 93.66	445.53 ± 85.20	5.60 ± 2.97	622.903	0.000	0.000	0.000	0.055
6 week	278.81 ± 112.88^**^	275.55 ± 103.28^*^	–	0.352	0.726			
*IL-4 (pg/ml)*								
baseline	22.77 ± 4.77	22.82 ± 5.74	0.016 ± 0.01	465.268	0.000	0.000	0.000	0.952
6 week	21.45 ± 4.42	21.26 ± 5.92	–	0.179	0.858			
*IL-6 (pg/ml)*								
baseline	15.01 ± 4.72	15.66 ± 7.29	0.385 ± 0.19	147.665	0.000	0.000	0.000	0.526
6 week	14.53 ± 6.09	14.76 ± 5.46	–	–1.192	0.848			
*IL-17 (pg/ml)*								
baseline	19.46 ± 6.13	19.05 ± 6.26	NA	0.319	0.751			
6 week	12.22 ± 5.82^*^	13.30 ± 5.58^*^	–	−0.913	0.364			

Between-group differences were performed using ANOVA; Between-group differences were performed using independent sample *T*-test. Within-group differences were examined using paired *T*-test. *P*1: For between celecoxib group and healthy group; *P*2: For between placebo group and healthy group; *P*3: For between celecoxib group and placebo group.

*P < 0.05, **P < 0.01, for between the 6-week time point and baseline.

**Fig. 4:**
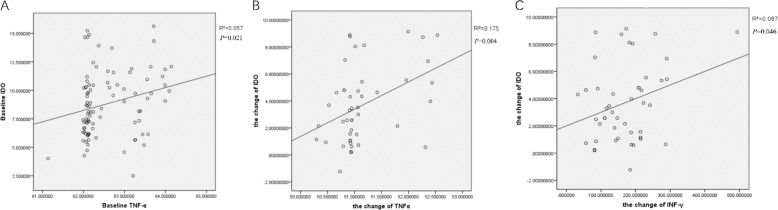
Correlation coefficients between IDO levels and TNF-α and INF-γ levels. **A** There was a significant positive correlation between serum IDO levels and serum TNF-α levels in all schizophrenia patients. **B** The change in IDO levels showed a significant positive correlation with the decrease in TNF-α levels in the celecoxib group. **C** The change in IDO levels showed a significant positive correlation with the decrease in INF-γ levels in the celecoxib group. *P* < 0.05.

In order to analyze the correlation between IDO and cytokines, we determined the association between IDO levels and cytokines levels at baseline, and also between the change in IDO levels and the change in cytokines levels before and after treatment. There was a significant positive correlation between serum IDO levels and serum TNF-α levels in all schizophrenia patients at baseline (*r* = 0.057, *P* = 0.021) (see Fig. 4A). The change in IDO levels showed a significant positive correlation with the decrease in serum TNF-α and IFN-γ levels in the celecoxib group (*r* = 0.175, *P* = 0.004; *r* = 0.087, *P* = 0.046) (see Fig. 4B, C). No correlation was found between IDO and other cytokines.

## Discussion

To our knowledge, this is the first study to examine peripheral IDO levels in first-episode drug-naive patients with schizophrenia and to explore the correlation between inflammatory molecules and IDO following treatment with the COX2 inhibitor, celecoxib. We had three major findings: (1) The elevated levels of IDO and inflammatory cytokines including IL-1β, IL-6, TNF-α, IL-17, IL-4, and IFN-γ can be observed in the early stages of schizophrenia; (2) IDO levels were significantly positively correlated with TNF-α and IFN-γ levels; (3) IDO levels were significantly positively correlated with negative symptoms of schizophrenia. These findings clarify the important role of IDO in the pathological mechanism of schizophrenia.

IDO is the initial and rate-limiting enzyme in the KP and can catalyze Trp into kynurenine. An increase in IDO expression or activity can accelerate Trp metabolism and promote the generation of downstream neuroactive metabolites. The kynurenine to Trp ratio (KYN/TRP) is a routinely used indicator of IDO activity. Previous studies have shown increased KYN/TRP in the plasma of patients with schizophrenia [29–31]. However, there have been no direct studies on IDO expression. In the present study, we found significantly increased IDO levels in first-episode drug-naive patients with schizophrenia compared to healthy controls, which is consistent with the results of previous studies. These findings suggest that abnormal activation of IDO occurs early in the disease and that the increase in IDO expression is related to the disease itself, not the drug treatment. Treatment with risperidone monotherapy or risperidone in combination with celecoxib both decreases IDO levels, indicating that both risperidone and celecoxib can be involved in the regulation of IDO expression. Schwieler et al. found that selective COX-2 inhibitors displayed an inhibitory action on the synthesis of endogenous rat brain KYNA [32]. Sung Yong Lee et al. found that COX-2 inhibitor celecoxib reduced IDO expression in 3LL tumor cells. And this inhibition is associated with T cells [28]. In another animal study, the researchers observed that celecoxib reverses the IFN-α-induced increase in the kynurenine/Trp ratio in rats [27]. In the present study, we found that celecoxib had a significant inhibitory effect on IDO expression. Although the methods and objects are different, the results of these studies all indicate that IDO may be a key node for COX2 inhibitors to participate in Trp metabolism and regulate downstream neuroactive metabolites of the KP. At present, there are no relevant studies on risperidone’s involvement in the regulation of IDO activity or expression. However, a large number of literature supports the anti-inflammatory properties of risperidone [11, 33–35]. For example, Zhang et al. revealed that risperidone reduced peripheral TNF-α levels in patients with schizophrenia [11]. Based on previous findings that pro-inflammatory cytokines can directly or indirectly increase IDO activity or gene expression, we speculated that risperidone may participate in the regulation of IDO expression through inflammatory factors, and then affect the KP. These views need to be tested in animal experiments.

As expected, the celecoxib-combined therapy had a significant effect on the improvement in PANSS negative symptom scores, as well as PANSS total scores and positive scores in first-episode drug-naive patients with schizophrenia. In a previous meta-analysis on augmentation with anti-inflammatory medications, we observed that some agents with anti-inflammatory properties, including aspirin, estrogens, minocycline, and N-acetylcysteine (NAC) showed efficacy for improvement of psychiatric symptoms [36]. However, the results of augmentation with celecoxib for schizophrenia treatment are heterogeneous. In a randomized, prospective, double-blind clinical trial, celecoxib demonstrated therapeutic efficacy when administered as an add-on treatment in patients with acute exacerbation of schizophrenia who were treated with risperidone [17]. Another clinical trial using celecoxib and amisulpride in patients with the first manifestation of schizophrenia also showed positive results [18]. In two trials on patients with continuously symptomatic schizophrenia, no advantage of celecoxib could be observed [17, 19]. Together with these data, we conclude that the effect of celecoxib depends on the duration of the disease, that is, the advantage of celecoxib is observed in patients with the first manifestation of schizophrenia or in patients with acute episodes.

In the present study, a significant positive correlation between IDO levels and the severity of negative symptoms was observed. Negative symptoms in schizophrenia, which can seriously impair functional ability, are often treated insufficiently by currently available antipsychotics. One of the possible mechanisms of negative symptoms is disruption of glutamatergic systems [11]. In a meta-analysis (*n* = 343) of 18 randomized placebo-controlled studies with glutamatergic drugs, Tuominen and colleagues found a mean reduction of four points on the PANSS-negative symptoms subscale [37]. Since one of the final neuroactive products of Trp metabolism is KYNA, which is the only endogenous NMDA receptor antagonist, we hypothesized that the mechanism by which IDO associates with negative symptoms is through the abnormal function of Trp metabolic pathways. Based on these findings, we are optimistic that agents regulating IDO such as celecoxib may be a new approach for the treatment of negative symptoms of schizophrenia.

Accumulating evidence suggests that dysregulations in components of the immune system are fundamentally linked to schizophrenia [38, 39]. Our findings of dysfunction of inflammatory cytokine levels in schizophrenia agreed with the previously mentioned cytokine-mediated immune disorders and demonstrated the anti-inflammatory properties of risperidone and celecoxib. A meta-analysis of 40 studies assessing cytokine alterations in acutely relapsed inpatients and those with first-episode psychosis found that some cytokines (IL-1β, IL-6, and transforming growth factor-beta (TGF-β)) may be state markers for acute exacerbations, while others (IL-12, IFN-γ, TNF-α, and sIL-2R) may be trait markers [40]. However, we observed inconsistent results in that there were significant decreases in serum levels of TNF-α, IL-1β, IFN-γ, and IL-17 after 6 weeks of treatment in first-episode drug-naïve patients with schizophrenia, while there was no change in levels of IL-4 and IL-6. In a 6-month follow-up study of first-episode drug-naive schizophrenia, Song et al. found that serum IL-1β, IL-6, and TNF-α levels changed dynamically throughout the follow-up period [41]. These heterogeneous results may be attributed to differences in observed time points.

Given the evidence that the activity of IDO is strongly regulated by the immune system and more specifically by pro-inflammatory cytokines, such as IFN-γ, IL-6, IL-1β, and TNF-α [42, 43], we predict that in our study, changes in pro-inflammatory cytokines levels described above would affect IDO expression. A recent study found a significant correlation between IFN-γ and kynurenine, and a trend-level correlation between TNF-α and kynurenine levels in plasma from patients with schizophrenia, but no significant correlation between IL-6 and kynurenine [44]. Another study in patients with schizophrenia reported a positive correlation between plasma IL-1β and kynurenine levels [45]. In this study of patients with first-episode schizophrenia and relatively well-matched controls, significant positive correlations between IDO levels and TNF-α and INF-γ levels were observed. These results supported the idea that pro-inflammatory cytokines including TNF-α and INF-γ have a positive regulatory effect on IDO.

In addition, anti-inflammatory drugs can play a role in the treatment of schizophrenia by regulating the expression and activity of IDO through inflammatory mechanisms.

The advantage of this study is the vertical observation of changes in peripheral blood IDO expression in patients with first-onset drug-naive schizophrenia, and the analysis of its association with peripheral blood cytokines and psychiatric symptoms. Compared with other previous studies, we studied the expression of IDO directly, instead of reflecting IDO activity via levels of downstream KP metabolites, such as kynurenine. Kynurenine itself is affected by many factors including smoking [46] and aerobic exercise [47]. However, there are also a few limitations. First, we only determined IDO expression in peripheral blood and did not determine the activity of IDO. Whether the change in expression levels can represent a change in an activity needs further verification. Second, with the exception of the IDO, we also need to determine the levels of other enzymes and neuroactive metabolites in the KP to fully understand the effects of pro-inflammatory and anti-inflammatory drugs on the KP. Third, we need to further examine the expression of IDO in the CNS. Although no studies have so far been able to confirm the consistency of central and peripheral IDO expression levels in patients with schizophrenia, our unpublished results of animal models of schizophrenia support the consistent trend of IDO changes in the two environments.

Despite these limitations, this study is the first to investigate the expression of IDO in peripheral blood of first-episode drug-naive patients with schizophrenia, and to determine the correlation between inflammation and IDO. Our results show that there is abnormally elevated IDO expression in patients with first-episode drug-naive schizophrenia, and IDO expression correlates with negative symptoms of schizophrenia and the levels of various pro-inflammatory cytokines in peripheral blood. These findings indicate the importance of IDO in the pathogenesis of schizophrenia.

## Supplementary information

aj-checklist
